# Fabrication, Mechanical Testing and Structural Simulation of Regenerated Cellulose Fabric Elium^®^ Thermoplastic Composite System

**DOI:** 10.3390/polym13172969

**Published:** 2021-08-31

**Authors:** Pooria Khalili, Mikael Skrifvars, Ahmet Semih Ertürk

**Affiliations:** 1 Swedish Centre for Resource Recovery, University of Borås, 510 90 Borås, Sweden; mikael.skrifvars@hb.se; 2 Department of Industrial and Materials Science, Chalmers University of Technology, 412 96 Gothenburg, Sweden; erturk@chalmers.se

**Keywords:** regenerated cellulose fibre, thermoplastic resin, finite element (FE), mechanical performance

## Abstract

Regenerated cellulose fibres are an important part of the forest industry, and they can be used in the form of fabrics as reinforcement materials. Similar to the natural fibres (NFs), such as flax, hemp and jute, that are widely used in the automotive industry, these fibres possess good potential to be used for semi-structural applications. In this work, the mechanical properties of regenerated cellulose fabric-reinforced poly methyl methacrylate (PMMA) (Elium^®^) composite were investigated and compared with those of its natural fibre composite counterparts. The developed composite demonstrated higher tensile strength and ductility, as well as comparable flexural properties with those of NF-reinforced epoxy and Elium^®^ composite systems, whereas the Young’s modulus was lower. The glass transition temperature demonstrated a value competitive (107.7 °C) with that of other NF composites. Then, the behavior of the bio-composite under bending and loading was simulated, and a materials model was used to simulate the behavior of a car door panel in a flexural scenario. Modelling can contribute to predicting the structural behavior of the bio-based thermoplastic composite for secondary applications, which is the aim of this work. Finite element simulations were performed to assess the deflection and force transfer mechanism for the car door interior.

## 1. Introduction

Pulp, paper and wood fibres, along with plant fibres, for instance, jute, flax and hemp, have attracted considerable attention in the past two decades. It has continually been appealing to utilise bio-based/natural fibres rather than synthetic ones, in particular glass fibres (GF) as the predominating reinforcing element [1].

Regenerated cellulose (rayon) fibres have been demonstrated to present substantial promise as reinforcements for thermoplastics [1,2,3]. Regenerated cellulose fibres possess unique characteristics in the sense that they offer the merit of both natural (NF) and synthetic fibres. These include on the one hand low density, CO_2_ neutrality [4], non-abrasiveness to processing equipment [4] and the biodegradability [5] of NFs, and on the other hand, the physical, mechanical and uniform morphological properties of synthetic ones. Rayon is manufactured by the regeneration of dissolved cellulose and is utilised in the textile yarn forms. Due to their high tenacity, which leads to good impact resistance, high specific strength and low density (~1.5 g/cm^3^), rayon yarns have been utilised to reinforce car tires as tire cord [6]. 

Several articles have been published on the topic, spanning a diversity of manufacturing routes and combinations of rayon fibre and matrices. Primarily, works have been performed to produce short-fibre thermoplastic composites using melt-mixing coupled with injection moulding, followed by solvent impregnation or pultrusion [1,3,7] and compression moulding for continuous fibre composites. Polypropylene (PP) seems to have been the most attractive choice of polymer for regenerated cellulose fibre-reinforced composites thus far. 

It was reported that short rayon fibre PP composites demonstrate comparable mechanical performance to those of GF PP materials at the same fibre mass fraction (30 wt%) [1]. Although the tensile modulus was lower for regenerated cellulose PP composite (2.9 GPa) than for its GF counterpart (4.1 GPa), the strength was measured at 78.7 MPa, which showed a 40% increase compared to the GF PP composite. This proves the potential of this type of bio-based fibre as a reinforcement in composite systems. The resulting rayon PP composite was utilised in the production of door panels and dashboards by Faurecia interior systems and Cordenka [7]. In the automotive industry, it was revealed that rayon-based composites offer excellent mechanical performance, reaching and partially surpassing the level of GF ones [8,9]. In comparison with NF counterparts, regenerated fibre composites display significant enhancement in strength and impact properties [7], whilst stiffness can easily be improved by the incorporation of NFs at a good level [10]. 

For semi-structural applications, these sorts of fibres can be used in the form of fabric, and the woven structure of the reinforcement can further enhance the properties of the developed composite. It is customary to use a type of liquid thermoset to develop a composite based on textile reinforcements. Thermosets, due to their high mechanical behaviour, are used in high-performance applications. On the other hand, thermoplastics are appealing to manufacturers as they provide high impact tolerance [11], good vibration-dampening capacities [12], recyclability [11] and post-manufacturing formability [13], and some have eco-friendly contents [14]. A novel thermoplastic resin (Elium^®^) has been developed by Arkema Chemicals, which enables the process of composites at room temperature (as a result, it is a less energy-intensive thermoplastic polymer relative to other ones) and offers similar mechanical properties as epoxy-based systems. This manufacturing condition contributes to preventing the degradation of the performance of the bio-based fibre, which is generally caused by the high processing temperature of thermoplastics. Resin infusion and resin transfer moulding (RTM) can be employed to polymerise the fibre-reinforced Elium^®^ part at ambient temperature. The incorporation of a peroxide curing agent into the monomer (methyl methacrylate (MMA)) initiates free radical polymerisation, which subsequently reacts to form a poly methyl methacrylate (PMMA) polymer [15,16]. It is worth noting that higher bio-based fibre mass fraction can be obtained in the composites when using hot compression or resin infusion methods compared to that which can be obtained from an injection moulding technique. This can be a crucial point that needs to be taken into account when the presence of maximum fibre content in the part is important [17,18,19].

In this work, the reinforcing capabilities of rayon fabric in a liquid thermoplastic resin were investigated. The mechanical performance and glass transition temperature of the resulting composite were compared with those of other bio-based and NF (jute and ramie) thermoset/thermoplastic composite systems. Very few studies reported the tensile, flexural and viscoelastic behaviour of rayon fabric Elium^®^ composite (the combination of a regenerated cellulose fabric and Elium^®^) and were found to show the potential of this type of bio-based fabric composite for secondary applications. Scanning electron microscope (SEM) analysis was performed to investigate the mode of fracture after the tensile test. Rayon fabric Elium ^®^ composite produced using the resin infusion (RI) method, which is typically employed in fabric-reinforced thermoset composites. Rayon and Elium^®^ were used for the first time to make a bio-based fabric thermoplastic composite using the RI method, to the best of the authors’ knowledge. A finite element (FE) model was developed, and the model was indicated to be capable of predicting the structural performance of the regenerated cellulose Elium^®^ composite panel. The bending performance of the bio-based composite was modelled with a polynomial function, and the materials model was applied to the FE (ABAQUS) software for the calibration and validation of the bent rectangular sample. Subsequently, the same materials model was assigned to a door car (interior) panel, and then the bending deformation, deflection and reaction forces were predicted. This kind of simulation has not been extensively carried out for bio-based composites.

## 2. Materials and Methods

### 2.1. Materials

Cordenka GmbH, Obernburg, Germany provided 0/90 plain-woven rayon fabrics with a nominal weight of 442 g/m^2^, which had a linear density and breaking force of 2485 dtex and 796 MPa, respectively. Elium^®^ 150 polymer was supplied by Arkema, Colombes, France. The viscosity and liquid density were 0.1 Pa·s and 1.01 g/m^3^, respectively, and dibenzoyl peroxide was used as curing agent.

### 2.2. Processing

The composite plates measuring 100 mm × 300 mm were produced using the resin infusion technique [2]. Three layers of rayon fabrics were dried in a convection oven for 24 h at 70 °C prior to the start of infusion. They were placed on a surface-coated mould, followed by positioning a layer of peel ply on the fibres. Spiral tube, silicon connectors, outlet PVC hose and resin feed PVC hose were connected to the assembly. Subsequently, the whole system was sealed with the aid of gum tape and a vacuum bag. As recommended by the manufacturer, to avoid the shrinkage of composites, double bagging was employed to seal the assembly. Then, the compaction of the fabrics was performed to enhance the fibre fraction in the resulting composite; the vacuum was applied in the vacuum bag and released thrice for this purpose. The sealed assembly was left for 10 min to ensure a leakage-free system. The resin and dibenzoyl peroxide at a stoichiometric ratio of 100 to 1.5 parts by weight were stirred manually for three min, and the mix was degassed for three min to eliminate the trapped gases. The resin infusion was carried out, and the part was left to cure for 24 h at the ambient temperature. The composite plates possessing about 1.5 mm thickness were demoulded, and the fibre volume and mass fractions were 50% and 55%, respectively. The infusion process lasted 4.5 min for three layers of fabrics at the mentioned dimensions. The resulting composites were cut into specified sample dimensions with the aid of laser cutting equipment for tests. 

### 2.3. Characterisation

The bending performance of the rayon fibre-reinforced composite laminate was studied using a Tinius Olsen H10KT universal testing instrument, Horsham, PE, USA, in accordance with BS EN ISO 14125. Five rectangular specimens with dimensions of 80 mm × 20 mm (length × width) and a span length of 64 mm were characterised. The cross-head speed and the load-cell of the test machine were fixed at 5 mm/min and 5 kN, respectively. For all types of tests in this work, the samples were conditioned for 24 h in a humidity chamber with a temperature of 23 °C and humidity of 50% before the characterisations. 

Tensile properties of the rayon fibre-reinforced composite laminate were investigated according to BS EN ISO 527 on a Tinius Olsen H10KT universal testing instrument, Horsham, PE, USA, equipped with a 100R mechanical extensometer. Five rectangular specimens measuring 150 mm × 20 mm (length × width) were tested, and both ends of specimens were attached to the tabs of 25 mm length. The load-cell and the cross-head speed were set to 5 kN and 1 mm/min, respectively.

A one-way statistical analysis was performed by ANOVA to investigate the variance for the tensile and flexural test results individually. The significant differences between the composites in their strength and modulus were investigated. To evaluate the significance of each property, the *p*-value parameter was introduced.

The cross-sectional surface of tensile specimens after fracture was investigated using scanning electron microscopy (SEM). The machine was a FEI Quanta200 ESEM (FEI microscopes, Hillsboro, OR, USA) operating an accelerating voltage of 3 kV. The specimens were gold sputtered on a sputter coater Edwards S150B, Perth, UK with plasma exposition of 60 s in vacuum before the scanning.

Dynamic mechanical analysis (DMA) tests were carried out with the aid of Rhemetrics Solids Analyzer RSA II, (TA Instruments, New Castle, DE, USA). The three-point bending mode was used for the samples previously conditioned for 24 h at 23 °C and 50% humidity in a humidity chamber prior to the testing. The dimensions of the rectangular samples was 50 mm × 10 mm, and the heating rate and frequency were set to 5 °C/min and 1 Hz, respectively. The temperature was increased from 30 °C to 170 °C, and subsequently, the storage modulus (E′) and tan δ (loss factor) were measured.

## 3. Results and Discussion

Tensile tests were performed to study the strength, modulus and elongation at break of the rayon Elium^®^ composite and to compare the respective results with the available natural fibre (NF) composite counterparts.

The incorporation of rayon instead of NF was seen to enhance the strength and toughness of the composites compared to that of natural fibre composites, as the breaking point elongated to a much higher value (Table 1) using the same manufacturing technique, as conducted by the authors [17,20]. It was shown that rayon fibre has higher elongation and lower modulus than flax fibre [21].

ANOVA analysis for the tensile strength of the above-mentioned composites is displayed in Table 2, which includes the variance of the tensile strength, i.e., between groups (BG) and within group (WG). SS refers to the sum of square, and df stands for degree of freedom. The F is the ratio of the BG mean square (MS) to the WG mean square. For all three composites, 5 samples were considered; therefore, there were 15 samples in total. If the *p*-value of the F-test is less than 0.05, as reported here, the difference between the average tensile strength from one type of composite to another at a 95% confidence level is statistically significant. Table 3 shows the ANOVA analysis of the tensile modulus; similarly, the P-value was calculated to be less than 0.05, indicating a statistically significant difference between the average tensile modulus from one composite to another at the confidence level of 95%.

It was detected that the rayon fibre treatments with acetylation, 3-methacryloxypropyltrimethoxy silane (MPS) and 3-aminopropyltriethoxy silane (APS) were found to reduce the tensile strength by 2.1–35% and modulus by 2–24% of the resulting composites; however, the elongation slightly increased only for MPS-treated rayon fibre unsaturated polyester composite, as measured by one of the authors before [22]. The transfer of stresses goes from matrix to fibre; thus, the fibre–matrix interface is very important but was not achieved in these investigations. As the tensile properties were more fibre-dependent, this explains the ineffectiveness of rayon fibre chemical modifications. 

**Figure 1:**
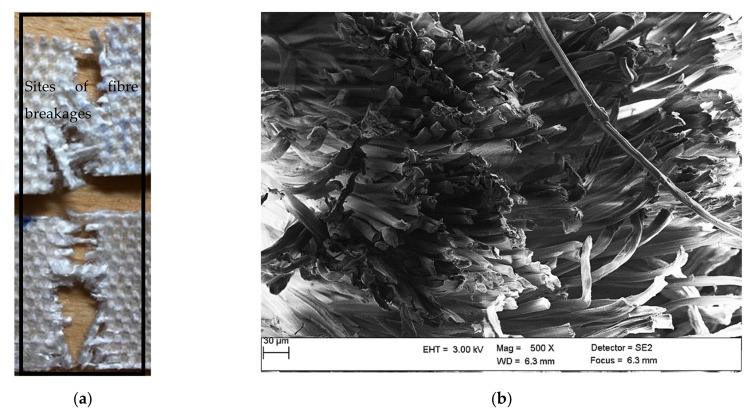
(**a**) Digital image and (**b**) cross-sectional SEM micrographs of the fractured rayon composite sample after tension at 500× magnification.

As illustrated in Figure 1, no indication of fabric delamination was detected in the cross-sectional image, and the mode of fracture was fibre breakage. The sites of fractures are shown in Figure 1a and fibres broke along the tension direction are displayed in Figure 1b. Good interfacial adhesion was observed between the rayon fibres and the Elium^®^ matrix.

**Figure 2:**
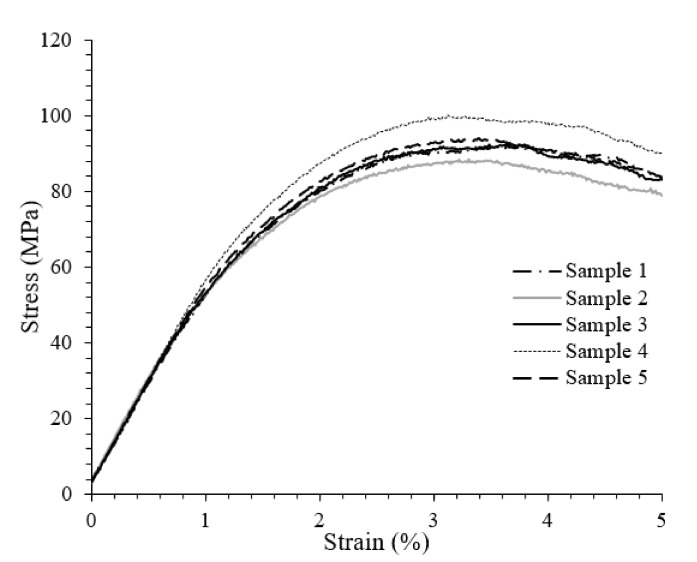
Stress–strain behavior of rayon composite samples.

The three-point bending strength and modulus of rayon fibre-reinforced Elium^®^ composite samples were measured in order to evaluate the properties and compare them with those of natural (ramie and jute) fibre Elium^®^ composite systems, as well as rayon fibre epoxy composites, fabricated via the same production method. The stress–strain curves of rayon fibre-reinforced Elium^®^ composite samples are displayed in Figure 2, and the average flexural strength and modulus are tabulated (Table 4). The values are 93.5 MPa and 5.6 GPa for strength and modulus, respectively, which are in the same range as rayon fibre epoxy composite (as described in the previous work) [23]. Moreover, 0&90 woven jute fabric Elium^®^ was found to show lower strength (7%) and modulus [20].

It was shown that the chemical treatment of rayon fibres did not lead to the enhancement of the flexural properties of their respective composites. For instance, γ-aminopropyltriethoxysilane (APTES) was used to modify the rayon fibre surface, and, in some composites, a silane-coupling agent was used for incorporation into the epoxy system; however, no improvement was recorded for the flexural performance of the developed rayon epoxy composites [23]. In another investigation by the author [22], 3-methacryloxypropyltrimethoxy silane (MPS) and acetic anhydride were employed for silane and acetylation treatments of the rayon surface to produce treated rayon unsaturated polyester composites, which did not provide positive results for bending modulus and strength. 

**Figure 3:**
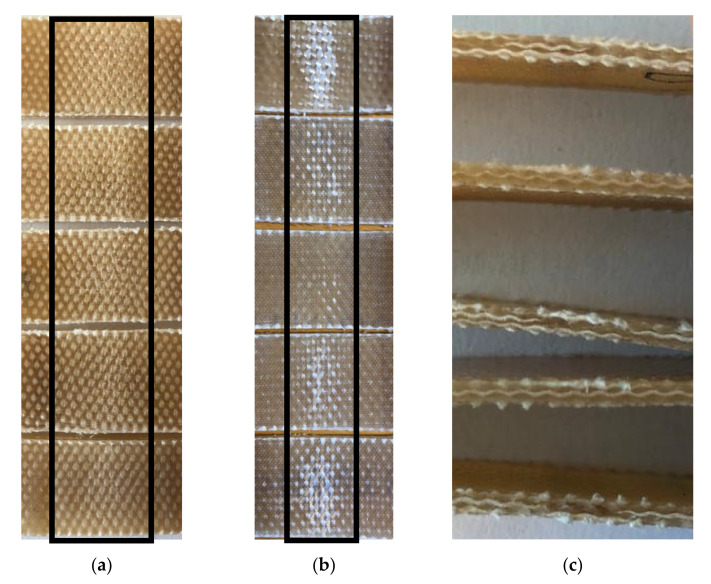
Digital images of (**a**) the middle of samples’ bottom surfaces having undergone tension, (**b**) the middle of samples’ top surfaces having undergone compression and a (**c**) cross-sectional view of the bent samples right in the middle after flexural tests.

It was observed that the bending samples did not fracture even after 7% bending strain; instead, very tiny cracks were observed on the tensile and compressive sides of the samples. On the tension and compression surfaces of the bent samples, the matrix became whiter and less transparent due to the breakage of polymer chains (Figure 3a,b). The cross-sectional views of the bent samples were detected to illustrate no signs of delamination or breakage, as depicted in Figure 3c.

Tabulated ANOVA analysis (Table 5 and Table 6) shows the variance of flexural properties for the rayon Elium^®^ and jute Elium^®^ composites. The variance of flexural strength calculated between the groups and within groups (each group contains the result of five samples) demonstrated that the *p*-value equals 0.05. This implies that the difference between the flexural strength of these two composites was relatively significant, at slightly less than a 95% confidence level. This value could be explained by the fact that the bending test is more matrix-dependent, and, as the same matrix was used to make these two composites, the results can partly overlap. However, a *p*-value of less than 0.05 was obtained from the F-test of the modulus values, which indicates a statistically significant difference between the average flexural modulus of these two composites at a confidence level of 95%.

Besides, in order to have a better understanding of the performance of the bio-based composite in an actual part, first finite element (FE) simulation of the bending scenario was calibrated and validated with the experiment, and subsequently, the materials model was applied to a car door panel for predicting the behavior of the component under flexural loading. 

**Figure 4:**
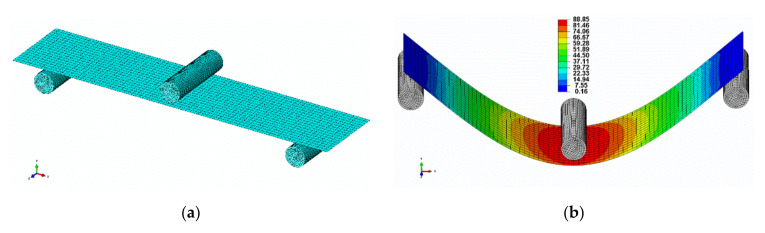
(**a**) FE model perspective of the bio-based composite sample and (**b**) the von Mises stress applied to the specimen.

The whole flexural setup was designed according to the experiment, as shown in Figure 4, and a materials model was adopted as per the experiment test result. The width, length and span of the sample were 20 mm, 80 mm and 64 mm, respectively, and it was modelled as a homogeneous shell. The supports and the load, which are presented as a cylinder with a diameter of 5 mm, were modelled as rigid. The modulus was obtained from the experiment and density, and Poisson’s ratios were 1.29 g/cm^3^ and 0.37 [24,25], respectively. Coulomb friction was used to model the friction between surfaces with a coefficient equal to 0.2 [26]. Polynomial materials modelling was assumed to model the plastic constitutive behavior. The bottom cylinders were fixed in all directions and rotations, whereas the top cylinder was fixed in the *x* and *z* directions and could only move downwards (along the *y* direction). The mesh type of the sample was a 2D shell quad with an element size of 1 mm, and the number of elements was 1440. The parameters above were found to be optimal in terms of computational time and accuracy convergence after performing some preliminary investigations of the element size. The FE model with the mesh and the color representation of the von Mises stress subjected to the bending load is displayed in Figure 4a,b and a vertical displacement of 18 mm was registered in the center of the sample. 

A polynomial function of degree two was used to estimate the plastic deformation of the sample and is obtained as

(1) $$\sigma =\sum_{k=0}^{n}a_{k}x^{k},$$

where *σ* is the stress (in MPa); *x* is the instantaneous strain; *a_k_* is the strain amplitude, and *k* is the strain exponent. Polynomial expression fitted the experimental results by using the strain amplitudes of *a*_2_, *a*_1_ and *a*_0_, which were employed as approximately 8, 54 and 10, respectively, and the strain exponent of *k* = 2. All of these parameters were extracted with a coefficient of determination (R2) of 99%. 

**Figure 5:**
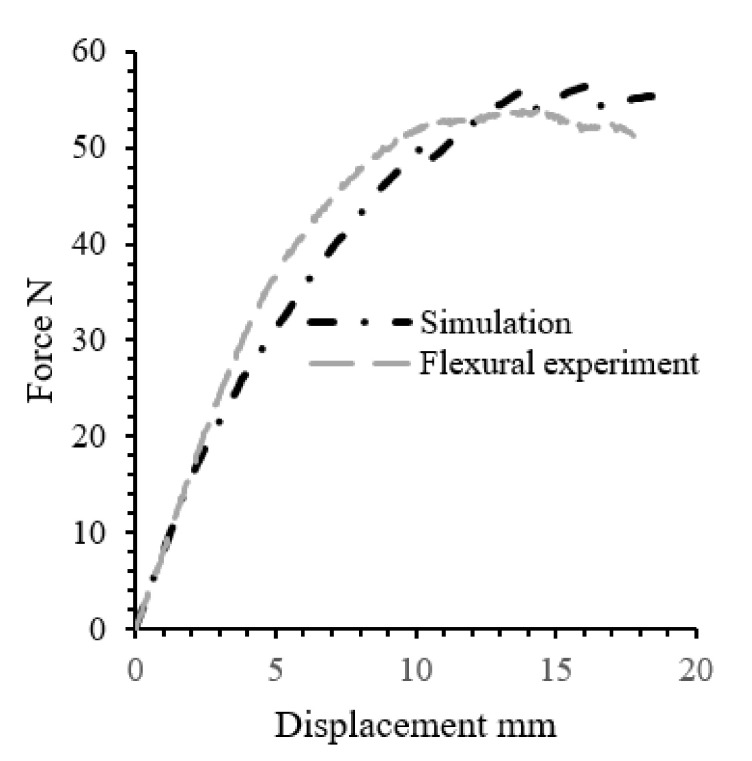
Force-displacement simulation of a three-point bending scenario.

A comparison between FE model results in terms of force-displacement and the experimental measurements is depicted in Figure 5. Generally, a good agreement was obtained between the experimental result and the FE simulation. The small deviation in the plastic region is due to the fact that the average Poisson’s ratio used for this simulation was the value obtained from the literature, which could not demonstrate the precise amount. This validated the developed model and enabled its utilisation for the prediction of the bending performance of the real-life component.

The simulation enabled the assessment of the displacement-force performance and the deformation of the car door panel made of the same developed material in a bending scenario. 

**Figure 6:**
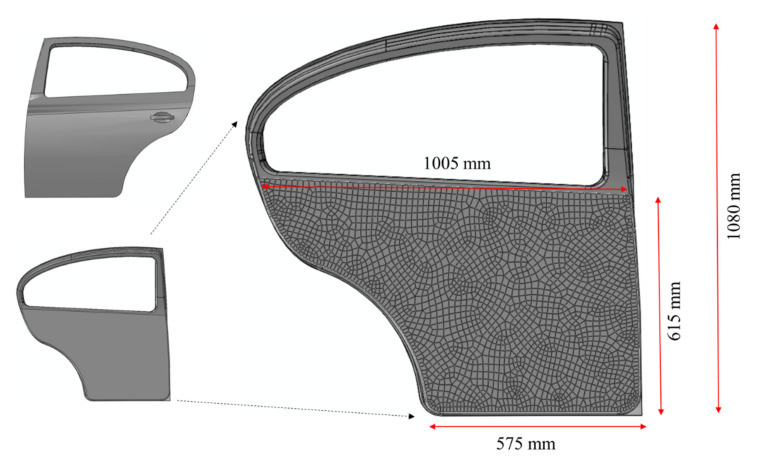
The mesh and dimension of the car door interior.

**Figure 7:**
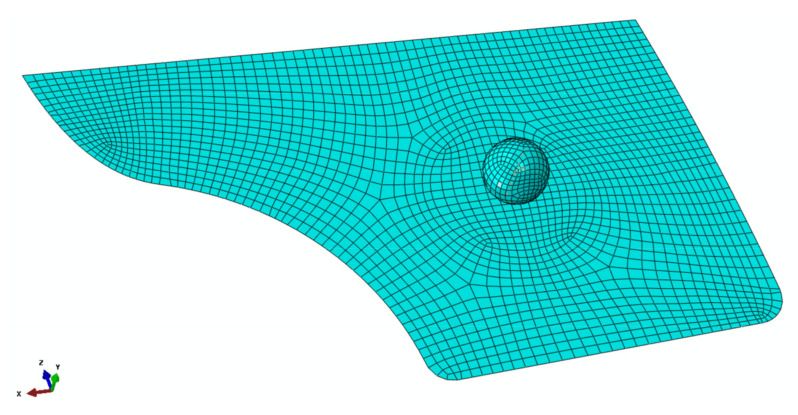
FE model perspective of the panel and the sphere.

**Figure 8:**
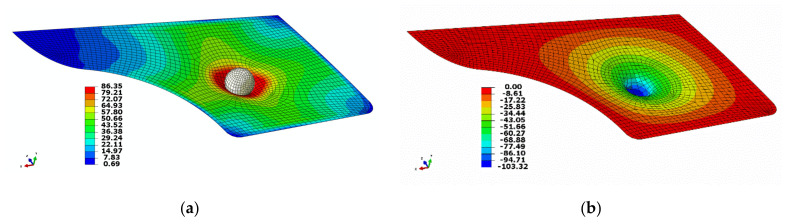
Deformed shape of the door interior. The color representation of (**a**) von Mises stress and (**b**) vertical displacement.

**Figure 9:**
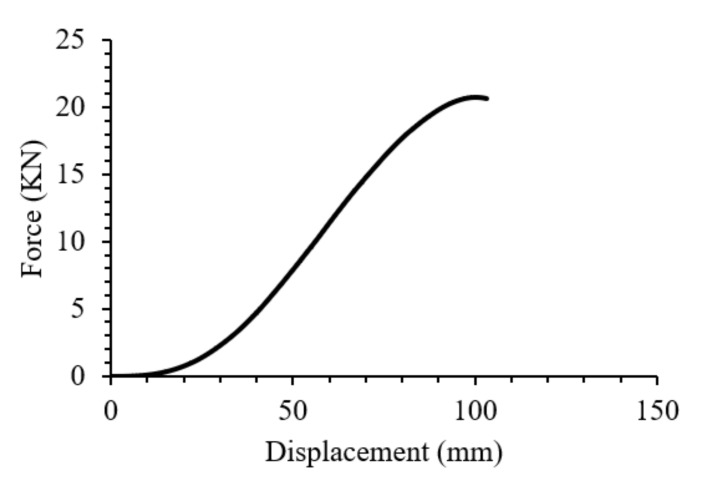
Prediction of the force-displacement behavior of the car door panel made of regenerated cellulose fabric Elium^®^ composite.

The swift car door panel [27] was used to simulate the bending of the part, which was modelled with the same materials model used for the bending test validation, under a flexural load in the shape of a sphere measuring 100 mm diameter. A friction coefficient of 0.2 was applied for the interaction between surfaces. The sphere placed in such a way to bend the middle of the door panel and its position from the bottom right corner of the door panel was 337 mm and 284 mm in the x and z directions, respectively. The dimension and mesh of the door interior are depicted in Figure 6. The FE model of the whole assembly, i.e., the sphere and the door panel is also shown in Figure 7. All the parameters were adjusted in accordance with the materials model developed in the flexural test experiment. The door panel was fixed from all the edges and the displacement to the *Y* direction was enabled in the assembly. When the sphere went down into the middle of the component, the maximum load was obtained at the deflection of around 100 mm (Figure 8b). However, the deflection could be much lower if the panel was supported by the door exterior. It is worth mentioning that the von Mises stress obtained (~86 MPa) was close to that of the bending test scenario (Figure 8a). Eventually, the prediction of the deflection-force behavior of the car door panel bent by the ball is shown in Figure 9. At around 102 mm deflection, a maximum load of approximately 20 kN could be recorded. This sort of materials modelling and structural simulation could contribute to the prediction of the performance and deformation of the automotive interior components, which can be fabricated using bio-based composites.

**Figure 10:**
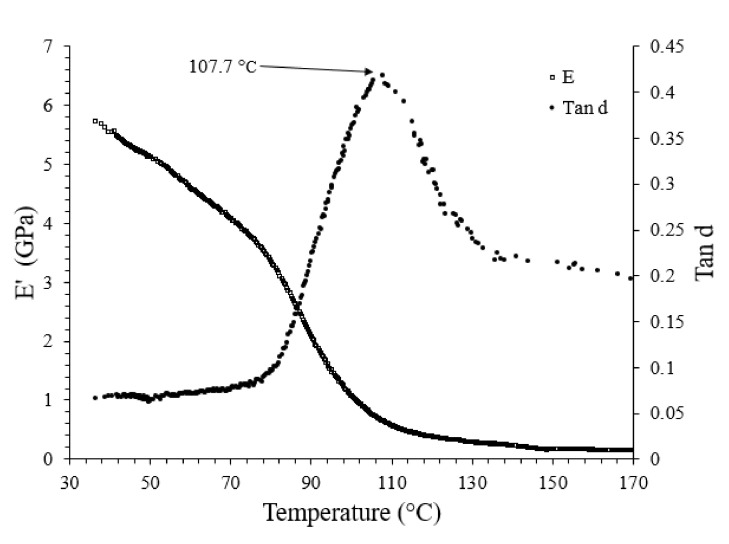
Average Tg as obtained from DMA analysis.

The DMA test was also performed to investigate the viscoelastic properties of rayon fibre Elium composite and to compare them with those of NF polymeric composites investigated by the authors, as shown in Figure 10.

The storage modulus (E′) was measured at 5.8 GPa at 30 °C, which is in good agreement with the flexural modulus as obtained from the three-point bending test at room temperature. The glassy state region lasted until 80 °C wherein glass transition phase was initiated, as illustrated in the E′ curve. The glass transition zone continued until about 100 °C, with a swift continuous drop in E′.

It can be discovered that the glass transition temperature of rayon fibre-reinforced Elium^®^ composite (107.7 °C), as obtained from the loss factor peak (tan (d)), is higher or comparable with that of rayon epoxy systems and other natural fibre Elium^®^ counterparts. The standard deviation of the T_g_ value for the composite is shown in parentheses in Table 7. 

The whole mechanical results and viscoelastic analysis demonstrate the great potential of the developed bio-based composite for the interior applications, and structural simulation can be performed to initially predict the mode of deformation and expected force in the component.

## 4. Conclusions

A series of experimental tests were conducted to evaluate the mechanical properties of a viscose-type fabric-reinforced thermoplastic composite. It was indicated that the developed material has good potential to be used for interior applications, which was one of the main aims of this study. In terms of strength, elongation and flexural properties, the regenerated cellulose fabric thermoplastic bio-composite demonstrated superior (or comparable) performance compared to the NF epoxy or Elium^®^ composite, whereas the Young’s modulus was found to be lower. It was indicated by one-way ANOVA analysis that the difference between the rayon Elium^®^ composite and the other counterparts for the tensile and flexural properties was statistically significant; however, for the flexural strength, the confidence level was slightly less than 95%. T_g_ was recorded at a suitable value of 107.7 °C, placing the developed bio-composite at a comparable level. Moreover, the polynomial material model was fitted, and it predicted the plastic deformation and forces subjected to the sample under the bending load in the ABAQUS software. The materials FE model was applied to a car door panel that was bent by a sphere measuring 100 mm diameter, and the deformation-force behavior of the part was predicted. 

The regenerated cellulose fabric thermoplastic composites reveal great potential for semi-structural applications, and this can be evaluated together with materials simulation before making the whole part or testing the bio-composite component.

## Figures and Tables

**Table 1:** Average tensile strength, modulus and elongation at break (EAB) of the rayon fibre composite compared to its NF counterparts.

	Tensile Strength (MPa)	STD (Strength)	Tensile Modulus (GPa)	STD (Modulus)	Elongation at Break (%)	STD (EAB)
Rayon Elium^®^ composite	77.5	2	3.2	0.2	10.2	1
Jute Elium^®^ composite [20]	46.7	3	7.9	0.5	4.7	0.4
Ramie Elium^®^ composite [17]	66	3	9.8	0.8	6.6	1.3

**Table 2:** ANOVA analysis for the tensile strength.

Source of Variation	SS	df	MS	F	*p*-Value
Between Groups	2429.33	2	1214.66	140.29	~0
Within Groups	103.89	12	8.65		

**Table 3:** ANOVA analysis for the tensile modulus.

Source of Variation	SS	df	MS	F	*p*-Value
Between Groups	121.52	2	60.76	237.41	~0
Within Groups	3.071	12	0.25		

**Table 4:** Comparative average flexural strength and modulus of rayon fibre composite.

Composites	Flexural Strength (MPa)	STD (MPa)	Flexural Modulus (GPa)	STD (GPa)
Rayon Elium^®^ Composite	93.5	3.8	5.6	0.4
Rayon epoxy composite [23]	93.7	~3.7	-	-
Jute Elium^®^ composite [20]	87.1	4.4	4.2	~0.1

**Table 5:** ANOVA analysis for the flexural strength.

Source of Variation	SS	df	MS	F	*p*-Value
Between Groups	101.41	1	101.41	5.29	0.05
Within Groups	153.33	8	19.16		

**Table 6:** ANOVA analysis for the flexural modulus.

Source of Variation	SS	df	MS	F	*p*-Value
Between Groups	4.06	1	4.06	34.46	0.00037
Within Groups	0.94	8	0.11		

**Table 7:** Comparative Tg values of Elium^®^ and epoxy composite systems with rayon and NF reinforcements.

Composite Samples	Tg
Rayon Elium^®^ Composite	107.7 (±0.2)
Rayon epoxy Composite [23]	~95
Raime Elium^®^ Composite [17]	99
Jute Elium^®^ Composite [20]	108.1

## Data Availability

Data shall be made available upon request.

## References

[1] Ganster J., Fink H.-P., Pinnow M.J.C.P.A.A.S. (2006). Manufacturing. High-tenacity man-made cellulose fibre reinforced thermoplastics–injection moulding compounds with polypropylene and alternative matrices. Compos. Part A Appl. Sci. Manuf..

[2] Khalili P., Kádár R., Skrifvars M., Blinzler B. (2021). Impregnation behaviour of regenerated cellulose fabric Elium^®^ composite: Experiment, simulation and analytical solution. J. Mater. Res. Technol..

[3] Ganster J., Fink H.-P. (2006). Novel cellulose fibre reinforced thermoplastic materials. Cellulose.

[4] Bledzki A.K., Gassan J. (1999). Composites reinforced with cellulose based fibres. Prog. Polym. Sci..

[5] Shibata M., Oyamada S., Kobayashi S.i., Yaginuma D. (2004). Mechanical properties and biodegradability of green composites based on biodegradable polyesters and lyocell fabric. J. Appl. Polym. Sci..

[6] Skrifvars M., Dhakal H., Zhang Z., Gentilcore J., Åkesson D. (2019). Study on the mechanical properties of unsaturated polyester sandwich biocomposites composed of uniaxial warp-knitted and non-woven viscose fabrics. Compos. Part A Appl. Sci. Manuf..

[7] Ganster J., Erdmann J., Fink H.-P.J.P. (2013). Biobased composites. Polimery.

[8] Weigel P., Ganster J., Fink H.-P., Gassan J., Uihlein K. (2002). Polypropylene-cellulose compounds-High strength cellulose fibres strengthen injection moulded parts. Kunstst. Plast Eur..

[9] Fink H.P., Ganster J. (2006). Novel Thermoplastic Composites from Commodity Polymers and Man-Made Cellulose Fibers. Proceedings of the Macromolecular Symposia.

[10] Khalili P., Tshai K., Kong I. (2017). Natural fiber reinforced expandable graphite filled composites: Evaluation of the flame retardancy, thermal and mechanical performances. Compos. Part A Appl. Sci. Manuf..

[11] Offringa A.R.J.C.P.A.A.S. (1996). Manufacturing. Thermoplastic composites—rapid processing applications. Compos. Part A Appl. Sci. Manuf..

[12] Chou P.J., Ding D., Chen W.-H. (2000). Damping of moisture-absorbed composite rackets. J. Reinf. Plast. Compos..

[13] Sadighi M., Rabizadeh E., Kermansaravi F. (2008). Effects of laminate sequencing on thermoforming of thermoplastic matrix composites. J. Mater. Process. Technol..

[14] Dordevic D., Necasova L., Antonic B., Jancikova S., Tremlová B. (2021). Plastic Cutlery Alternative: Case Study with Biodegradable Spoons. Foods.

[15] Van Rijswijk K.v., Bersee H. (2007). Reactive processing of textile fiber-reinforced thermoplastic composites—An overview. Compos. Part A Appl. Sci. Manuf..

[16] Bhudolia S.K., Perrotey P., Joshi S.C. (2017). Optimizing Polymer Infusion Process for Thin Ply Textile Composites with Novel Matrix System. Materials.

[17] Khalili P., Blinzler B., Kádár R., Blomqvist P., Sandinge A., Bisschop R., Liu X. (2020). Ramie fabric Elium^®^ composites with flame retardant coating: Flammability, smoke, viscoelastic and mechanical properties. Compos. Part A Appl. Sci. Manuf..

[18] Pisanu L., Santiago L.C., Barbosa J.D.V., Beal V.E., Nascimento M.L.F. (2021). Effect of the process parameters on the adhesive strength of dissimilar polymers obtained by multicomponent injection molding. Polymers.

[19] Fajardo Cabrera de Lima L.d.P., Santana R.M.C., Chamorro Rodríguez C.D. (2020). Influence of coupling agent in mechanical, physical and thermal properties of polypropylene/bamboo fiber composites: Under natural outdoor aging. Polymers.

[20] Khalili P., Blinzler B., Kádár R., Bisschop R., Försth M., Blomqvist P.J.M. (2019). Flammability, Smoke, Mechanical Behaviours and Morphology of Flame Retarded Natural Fibre/Elium^®^ Composite. Materials.

[21] Adusumali R.B., Reifferscheid M., Weber H., Roeder T., Sixta H., Gindl W. (2006). Mechanical properties of regenerated cellulose fibres for composites. Proceedings of the Macromolecular Symposia.

[22] Rajan R., Riihivuori J., Rainosalo E., Skrifvars M., Järvelä P. (2014). Effect of viscose fabric modification on the mechanical and water absorption properties of composites prepared through vacuum infusion. J. Reinf. Plast. Compos..

[23] Rajan R., Rainosalo E., Ramamoorthy S.K., Thomas S.P., Zavašnik J., Vuorinen J., Skrifvars M. (2018). Mechanical, thermal, and burning properties of viscose fabric composites: Influence of epoxy resin modification. J. Appl. Polym. Sci..

[24] Radzuan N.A.M., Tholibon D., Sulong A.B., Muhamad N., Che Haron C.H. (2020). Effects of High-Temperature Exposure on the Mechanical Properties of Kenaf Composites. Polymers.

[25] Fajrin J., Zhuge Y., Bullen F., Wang H. (2016). Flexural behaviour of hybrid sandwich panel with natural fiber composites as the intermediate layer. J. Mech. Eng. Sci..

[26] Abdolpour H., Garzón-Roca J., Escusa G., Sena-Cruz J.M., Barros J.A., Valente I.B. (2016). Development of a composite prototype with GFRP profiles and sandwich panels used as a floor module of an emergency house. Compos. Struct..

[27] https://grabcad.com/library/swift-car-door-shape-design-1.

